# Relationship of Dopamine of the Nucleus Accumbens with Intra-infralimbic Apomorphine Microinjection 

**Published:** 2013-06

**Authors:** Abbas Alimoradian, Javad Sajedianfard, Faegheh Baha-aldini Beigy, Mohammad Reza Panjehshahin, Ali Akbar Owji

**Affiliations:** 1 Department of Pharmacology, School of Medicine, Arak University of Medical Sciences, Arak, Iran; 2 Department of Physiology, School of Veterinary Medicine, Shiraz University, Shiraz, Iran; 3 Department of Pharmacology, School of Medicine, Shiraz University of Medical Sciences, Shiraz, Iran; E-mail: bzarandi@hotmail.com; 4 Natural & Medicinal Chemistry Research Center and Department of Pharmacology, School of Medicine, Shiraz University of Medical Sciences, Shiraz, Iran; 5 Department of Biochemistry, Shiraz University of Medical Sciences, Shiraz, Iran

**Keywords:** Apomorphine, Dopamine, Microdialysis, The nucleus accumbens, The prefrontal cortex

## Abstract

***Objective(s):*** The dopamine level of the nucleus accumbens changes during some stereotyped behaviors. To study dopamine level of the nucleus accumbens in intra infralimbic apomorphine-induced climbing, microdialysis probes were implanted into the nucleus accumbens shell of male Sprague Dawley rats weighting 275–400 g.

***Materials and Methods: ***The rats were divided into two groups (apomorphine and control) of least eleven rats in each group. Apomorphine at dose of 5 μg/0.5 μl or its vehicle was microinjected into the infralimbic in apomorphine and control groups respectively. Then, changes in dopamine levels in the nucleus accumbens shell were monitored. The concentration of dopamine was measured by High-Performance Liquid Chromatography-Electochemical (HPLC-ECD). Finally, the stereotyped behaviors were recorded.

***Results: *** The mean of dopamine levels for all of after microinjection period in control and drug groups were 450% and 150% respectively compared to those of before microinjection period. However, there was no significant difference between groups of apomorphine and control. In addition, the return of dopamine level to the baseline was faster in apomorphine group than the control group.

***Conclusion: ***The intra infralimbic apomorphine -induced climbing at dose of 5 μg/0.5 μl was not modulated via the increase of dopamine level in the nucleus accumbens area.

## Introduction

The pathological changes in the prefrontal cortex are involved in schizophrenia (1-4). Dysfunction of mesocortical dopamine inputs to the prefrontal cortex may underlie positive and negative symptoms associated with the schizophrenia (1, 5-7).

The medial prefrontal cortex is one of the regions of the prefrontal cortex involved in higher cognitive functions (4, 8, 9). The medial prefrontal cortex is not a homogenous structure (4, 8, 10) and can be subdivided into at least three subareas: the infralimbic, prelimbic and anterior cingulated with different intrinsic organizations, functions and distinct afferent- efferent connections (4, 11). 

The dopamine transmission of nucleus accumbens integrates a wide range of limbic and motor information (12-18). Based on the phasic dopamine transmission, the nucleus accumbens is subdivided into two heterogeneous compartments of shell and core (12, 19-21). 

Due to the importance of the medial prefrontal cortex in the pathogenesis of schizophrenia, the pathophysiologic study models have focused on the cortical dysregulation on subcortical dopamine neurotransmission (22). In addition, since microinjection of a dopamine agonist such as apomorphine into the brain area is a straight approach for investigation of the role of each area in stereotyped behaviors. 

It has been previously shown that the microinjection of apomorphine in the infralimbic could induce climbing significantly at dose of 5 μg/0.5 μl (23). Therefore, in this study we investigated the behavioral effects of microinjection of apomorphine in three subareas of the medial prefrontal cortex.

It is important to know whether induction of climbing via microinjection of apomorphine is a behaviors related to the medial prefrontal cortex or mediated by dopamine transmission of the nucleus accumbens via its relation with the medial prefrontal cortex. In the current study, apomorphine was therefore microinjected into the infralimbic subarea of the medial prefrontal cortex and released dopamine was measured by microdialysis method in the shell of nucleus accumbens to investigat if there was any possible involvement of the shell of nucleus accumbens in intra infralimbic apomorphine -induced climbing.

## Materials and Methods

***Ethics and Animals***

The protocol used in this study was approved by the Ethics Committee of Shiraz University of Medical Sciences. Eleven male Sprague Dawley rats (275–400 g) free access to food and water were used in each group for the experiments. 

***Drugs and Materials***

Apomorphine, 3, 4-dihydroxyphenylalanine (DOPA), Dopamine, Mandelic Acid and Tyrosine (all of them, Sigma, USA), 

Ascorbic acid, calcium chloride, glucose, magnesium sulfate, potassium chloride, sodium bicarbonate, sodium chloride, sodium dihydrogen phosphate and sodium hydroxide (all of them, Merck, Germany), 

Dialysis membrane, cut-off =6000D, fused silica connecting tube with Inner diameter (ID) =75 μm andouter diameter (OD) =150 μm (Eicom Company, Japan), Acetonitrile (Caledon, Canada), ketamine (Rotexmedica, Germany), xylazine (Alfasan, Netherlands), perchloric Acid (Hopkin & William, England), polyethylene tubing 10 and 20 (Stoelting, USA).

***Preparation of microdialysis probe***

Microdialysis probes were generated based on the Sharp and Zetterstrom method under stereomicroscope (Ziss, Germany) (24, 25). The dialysis membrane was left as much as 2 mm as the active size free to expose to the nucleus accumbens shell (26-32). Since the dialysis probes were "custom-made" some qualifications tests were performed. Final probes were then tested before the implantation in the rat. In this manner, the ability of probes for recovery of dopamine was determined as the *in vitro* environment. Their recovery was about 25%. 

***Implantation of probe and guide cannula***

 The rats were anesthetized by Ketamine + Xylazine (60 and 8 mg/kg respectively, IP intraperitoneal). The guide cannulas and probes were then implanted in the infralimbic and the nucleus accumbens shell respectively by stereotaxis (Stoetling, Wood Dale, IL, USA). The coordinate of the infralimbic was (AP, +3.2; L, +0.7; DV, - 5.4) and for probes into the nucleus accumbens shell at angle 15° from vertical was (AP, +1.7; L, +1.2; DV, - 8.2) (33). 

***Microinjection, microdialysis and behavioral recording***

The microdialysis experiments were performed 24 hr after the surgery. Apomorphine at dose of 5 µg/0.5 µl and 0.5 µl ascorbic acid 0.1% (as solvent of apomorphine and stabilizer) was delivered into the infralimbic as drug group and control or vehicle group respectively. The stereotyped behaviors were recorded as well (23) followed by the perfusion of probes with artificial cerebrospinal fluid (ACSF) at a flow rate of 2 μl/min (15, 34, 35) by microinjection pump (model 22, Harvard Apparatus, USA). In the next step, the fractions were collected in Eppendorf tube prefilled with 10 μl of perchloric acid 0.1mol as stabilizing agent (25, 36) each 20 min (15, 34, 37).

The compositions of ACSF include (in mmol) NaCl 114, KCl 3, CaCl_2_ 1, MgSO_4_ 2, NaH_2_PO_4_ 1.25, NaHCO_3_ 26, NaOH 1 and Glucose 10, pH 7.4 (38).

Dialysis procedure was 180 min divided into two parts, 60 and 120 min as before and after microinjections (34, 37). The samples were finally stored at -80°C until analysis by High-Performance Liquid Chromatography-Electochemical (HPLC-ECD).

***HPLC-ECD analysis***

**Figure 1:**
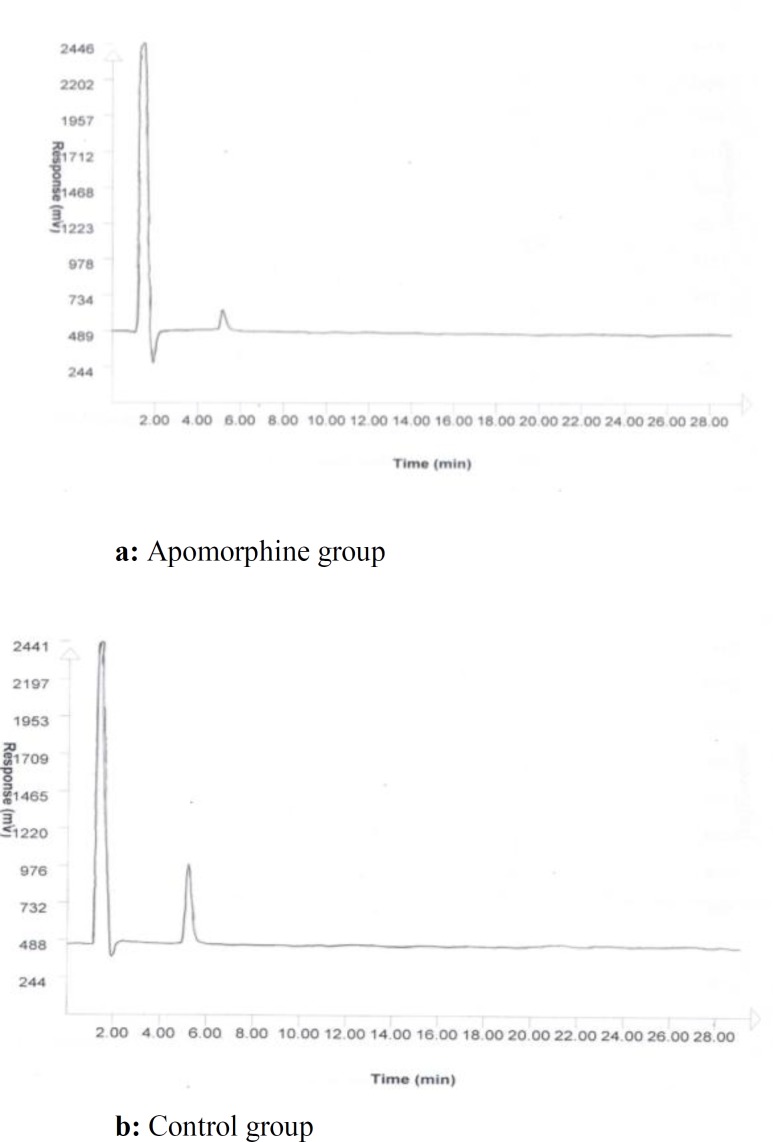
A chromatogram of a sample from apomorphine group (a) and control group (b) after microinjection

**Figure 2:**
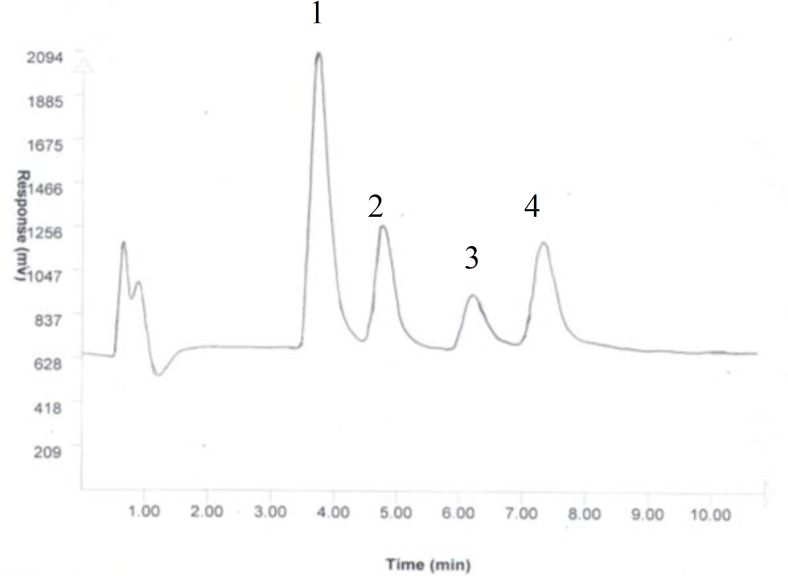
The chromatogram of peaks of some probable metabolites were detected by HPLC in a mixture of standards

The collected samples were analyzed by HPLC-ECD (model 460, Waters, USA). The mobile phase consisting of 100 mmol NaH_2_PO_4_ in HPLC grade water and 1% acetonitrile (pH3) was delivered into the reverse phase column (Reprosil-Pur C18-AQ 250 mm×3 mm ID, Dr A Maisch, Germany) at a flow rate of 1 ml/min (39, 40) The used method for microdialysis experiments and HPLC including microdialysis membrane cutoff, mobile phase and column were specific to the detection of dopamine in samples and those that dopamine metabolites or other neurotransmitters were not observed by HPLC detectors (Figure 1). However; in order to show if the aforementioned methododology has the ability to separate and detect any probable metabolites in samples, one standard artificial mixture of dopamine and its metabolites were injected into HPLC column (Figure 2).

The analysis of chromatograms was done by the Peak track software (Peak track, Capital HPLC Limited, England). The area under the curve (AUC) of standard solutions was used for the comparison surement of dopamine concentrations in the samples.

1: DOPA; 150 ng/ml, Retention time: 3-4 min

2: Dopamine; 150 ng/ml, Retention time: 4.5-5.5 min

3: Tyrosine; 10 μg/ml, Retention time: 6.5 min

4: Mandelic acid: 150 ng/ml, Retention time: 7-8 min

Volume of injection: 20 μl of mixture of standards

***Statistical analysis***

The analysis of data was performed by SPSS ver. 13. The data were analyzed by two and one way repeated-measures ANOVA using time as a repeated factor followed by LSD (Least Significance Difference) test as post hoc. The Significance level was set at *P*<0.05.

***Histological verification***

**Figure 3:**
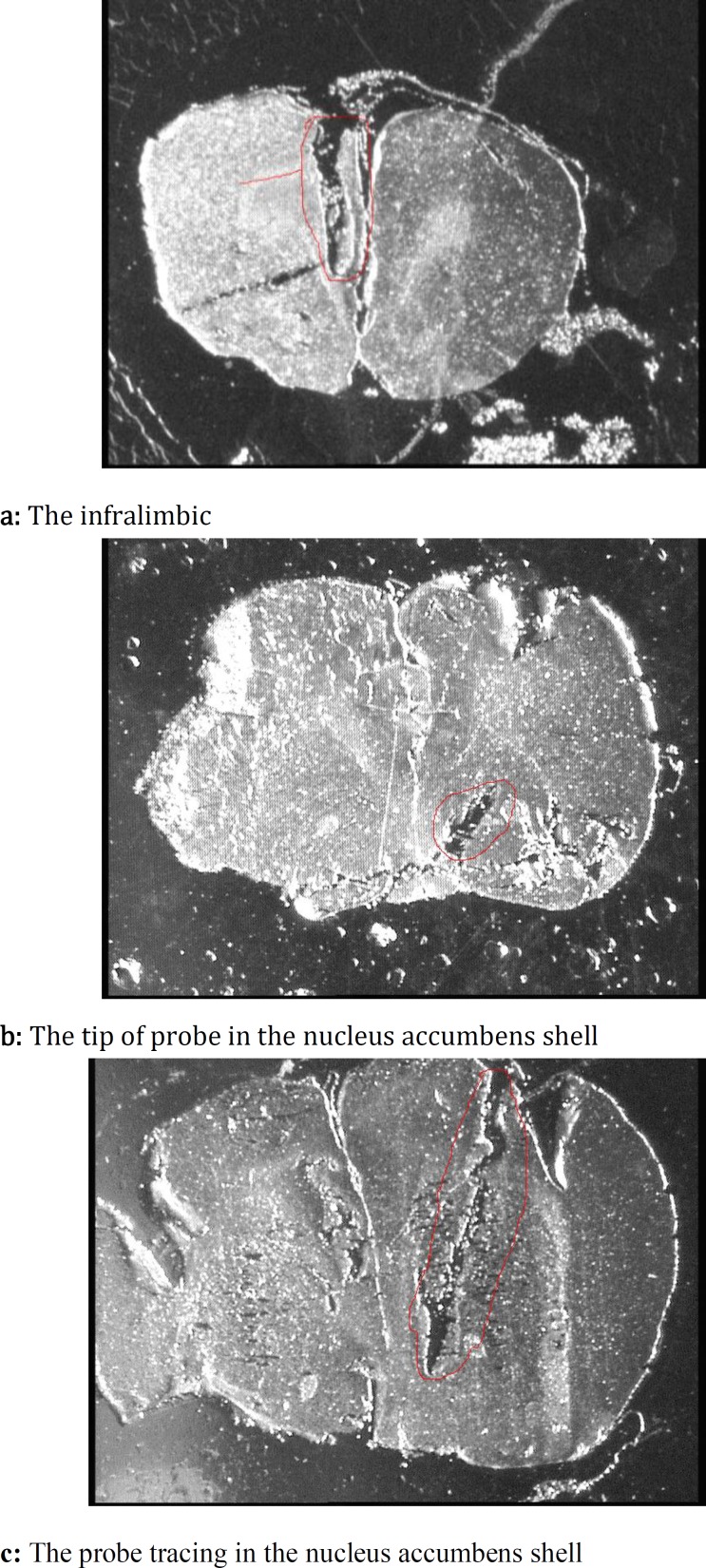
Stereomicroscopic photos of coronal section for cannula tracing at the level of the infralimbic (a), tip of probe in the nucleus accumbens shell (b) and probe tracing in the nucleus accumbens shell (c)

At the end of each test, serial sections were provided coronally by a microtome (model 1512, Leitz, Germany) and the positions of cannula and probe tracing were compared with the rat brain atlas (33) (Figure 3).

## Results

**Figure 4:**
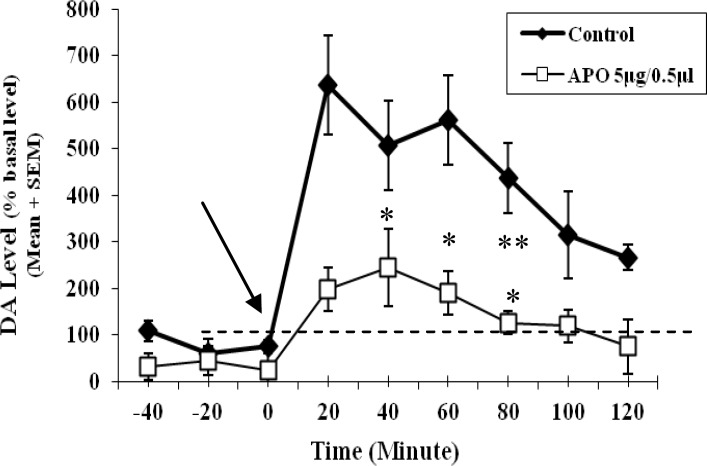
Time-course graph of changes of percent of extracellular dopamine level in the nucleus accumbens shell during 180 min

In this study, the mean of dopamine concentration of three samples prior to the microinjection period was considered as baseline level for each probe. The percentage of changes of dopamine concentration of samples during after microinjection period was estimated related to its own baseline level. The time-course graph presents the percentage of changes in extracellular dopamine from the baseline level (Figure 4). It was observed that the baseline values for the Apo morphine group was a little below 100% of the mean basal value. Nevertheless, it was concluded that it is quite obvious that it doesn’t matter though related to the normal variation of this physiologic parameter. Two way repeated-measures ANOVA using time as a repeated measure factor showed that the difference among dopamine concentrations of samples before any microinjection was not significant (F (2, 42) = 0.02; NS).

Two way repeated-measures ANOVA using time as a repeated measure factor showed that the parameter of time was significant (F (8, 168) = 5.084; *P*<0.01). However, there was not any significant difference between two groups of apomorphine and control (F (1, 21) = 0.259). There was not any significant interaction between time and treatment (control and apomorphine) factors (F (8, 168) = 0.508).

Each point represents the mean of percent of extracellular dopamine level related to the mean of basal level ±SEM

Each line represents time-course graph of percent of dopamine level for each group

Control: Ascorbic acid 0.1% as apomorphine vehicle; 0.5 μl microinjected into the infralimbic

APO: Apomorphine (5μg/0.5μl/rat); microinjected into the infralimbic

DA: Dopamine

Arrows show time of microinjection

Dotted line shows the mean of basal level

Before microinjection period: -40, -20^th^ minute and zero minute marked on horizontal axis

*: Significant at compared to the ninth 20 (120^th^ minute) minutes

**: Significant at compared to the seventh (80^th ^minute) and ninth 20 (120^th ^minute^)^ minutes

N=11-12 in each group

*P*<0.05

One-way-repeated measures ANOVA for each group of control or apomorphine using time as a repeated measure factor showed that factor of time was not significant for the control group, but it was significant for apomorphine group (F (3, 34) = 2.857;* P*<0.05). 

LSD test as *post hoc* revealed that there were significant differences in changes of percent of dopamine level among the fourth (20^th^ min), fifth (40^th^ min) and sixth (60^th^ min) 20-min episodes with ninth 20-min episode (120^th^ min), also between sixth (60^th^ min) and seventh 20-min (80^th ^ min) episode (*P*<0.05) (Figure 4). In addition, no significant stereotyped behavior such as climbing due to the microinjection of apomorphine was observed.

## Discussion

The nucleus accumbens (ventral striatum) is a heterogeneous area and has been subdivided into two different compartments, a medioventral shell and a laterodorsal core. The nucleus accumbens shell is involved in the control of motivation, emotional responses and most functionally reactive to the stress and abused drugs (21, 41). 

Furthermore, dorsal and ventral areas of the medial prefrontal cortex preferentially innervate the core and shell of nucleus accumbens respectively (42). Therefore, the infralimbic projects preferentially to the nucleus accumbens shell (4, 8, 43-45) and this subarea of the medial prefrontal cortex affect on dopamine release in the nucleus accumbens shell negatively (2, 46, 47). The different behavioral effects of microinjection of some doses of apomorphine into three subterritores of the medial prefrontal cortex were previously studied (23). The behavior of significant climbing was observed at the dose of 5 μg/0.5 μl of apomorphine after its microinjection into the infralimbic subarea of medial prefrontal cortex. The microdialysis study was performed to study the changes of dopamine level in the nucleus accumbens at this dose of apomorphine. 

In the present study, it was observed that the microinjection of either apomorphine or ascorbic acid 0.1% as apomorphine vehicle into the infralimbic subarea significantly increases the dopamine level in the nucleus accumbens shell (Figure 4).

Apomorphine as a nonselective dopamine agonist is a non-specific dopamine agonist for dopamine D1 and dopamine D2-like receptors (48, 49). Moreover, dopamine releases in the nucleus accumbens is affected by many factors such as abused drugs, stress may be regulated by other brain areas such as the prefrontal cortex. Handling is one of factors stimulating dopamine transmission and its release in the nucleus accumbens (50). In the present study, rats were handled gently for microinjection by Hamilton syringe during microdialysis experiments. This minimum handling would induce stress in free behaving rats and could induce dopamine release in the nucleus accumbens shell sensitive to the external emotional stimulants such as handling-stress. 

It has been reported that the increase of dopamine transmission in the ventral or deeper parts of medial prefrontal cortex including the prelimbic and infralimbic area has an inhibitory effect on turn-over of dopamine transmission in the mesolimbic system and its response to stimulants (4, 10, 22, 26, 27, 51, 52-57). The behavioral and neurochemical responses of the nucleus accumbens shell *e.g* locomotor activity and changes of dopamine concentrations to stress and stimulants are dampened by the concurrent activation of meso-prefrontal cortex dopamine neurons (26, 42, 55, 58). Therfore, dopamine depletion in the medial prefrontal cortex has potentiated stress-induced increase in extracellular dopamine in the nucleus accumbens shell (59). Thus, the reduction of dopamine levels in the prefrontal cortex increases dopamine release in the nucleus accumbens, whereas an increase dopamine levels in the prefrontal cortex attenuates the nucleus accumbens dopamine release (27, 36, 51, 55, 60-62). This inhibitory effect of the medial prefrontal cortex dopamine is directly via inhibition of excitatory amino acid neurons projecting to subcortical sites and indirectly by dopamine -mediated increases in γ-aminobutyric acid (GABA) release (56). The neurochemical and electrophysiological findings show that dopamine in the medial prefrontal cortex generally increases spontaneous GABA release and spontaneous firing of GABAergic neurons. Therefore, the medial prefrontal cortex dopamine exerts a (tonic) stimulatory effect on GABAergic interneurons thereby inhibiting glutamatergic pyramidal cells projecting to the nucleus accumbens and ventral tegmental area (4). It is worth mentioning that, dopamine depletion decreases the activity of GABA interneurons and triggers the activity of efferents to the nucleus accumbens including the reactivity of the mesolimbic dopaminergic system to environmental stimuli (1, 10, 26, 27, 37, 50, 55, 58). Therefore, the aforementioned reports are in consistent with the present finding (Figure 4) which depicts an increase of dopamine release in the nucleus accumbens shell. Handling stress due to the microinjection was less in drug group which in rats the received 5µg/0.5µl of apomorphine compared to the control group that the rats received the vehicle. The mean of dopamine level for all of after microinjection period in control and drug groups was 450% and 150% respectively compared to those of before microinjection period. The increase of dopaminergic activity in the infralimbic by apomorphine-microinjection at dose of 5 µg/0.5 µl as dopamine agonist decreased the nucleus accumbens shell response as dopamine release to the handling stress microinjection compared to the of control group. It has been reported that changes in dopamine neurotransmission in the prefrontal cortex do not have any effect on baseline of dopamine activity in subcortical dopamine systems such as the nucleus accumbens (1, 2, 22, 63, 64). Thus, amphetamine injections into the medial prefrontal cortex were found to reduce hyperlocomotion induced by intra-nucleus accumbens injections of amphetamine without producing an effect on locomotion by itself (4). These reports are also consistent with the present data showing no significant difference between the response of drug and control groups. Because the rats were handled very gently for microinjection, procedure and this handling-induced stress was not as effective as powerful environmental stimuli that could induce significant difference between the drug and control groups. As a result, changes in dopamine neurotransmission in the prefrontal cortex by apomorphine microinjection could not change dopamine release in the nucleus accumbens significantly. 

The findings of this study with no significant change in the dopamine neurotransmission of the nucleus accumbens shell after microinjection of apomorphine into the infralimbic vs. control group can also confirm the results of previous reports. With respect to another study (23), it seemed that the effect of apomorphine-microinjection into the infralimbic at dose of 5 μg/0.5 μl on behaviors such as climbing was a direct effect is not via the nucleus accumbens system. Thus, apomorphine at this dose induced climbing directly through the stimulation of dopamine receptors in the infralimbic and its effect was not related to the modulation of subcortical systems. If the effect of microinjection of apomorphine into the infralimbic at dose of 5 μg/0.5 μl on climbing was an indirect effect and related to the mesolimbic dopaminergic system. This thefore would induce a significant increase in dopamine release in the nucleus accumbens shell. Since these significant changes in dopamine activity of the mesolimbic system were not observed, the effect of apomorphine-microinjection into the infralimbic in previous report (23) at dose of 5 μg/0.5 μl on behaviors such as climbing could be a direct effect related to modulation of dopamine receptors of the infralimbic subarea and not via the nucleus accumbens. 

The changes of dopamine release after microinjection were significant in the drug group. The microinjection-handling stress has not been excessively influential to induce any significant differences of dopamine release between two groups in the nucleus accumbens, but because of the inhibitory effect of dopamine in the medial prefrontal cortex on dopamine neurotransmission of the nucleus accumbens, the return of dopamine level to baseline near to its level before microinjection was faster in drug group than that of control group (Figure 4). The increase of dopaminergic activity in the infralimbic by apomorphine microinjection may cause an increase of dopamine release in the nucleus accumbens in drug group. This did not last so long after microinjection and compared to the control group was significantly decreased to the baseline level faster.

Microdialysis probes could not keep their dialysis ability more than 24h after implantation (25); therefore, dopamine collection should be performed only 24 hr after the surgery. No significant stereotyped behaviors were observed in climbing due to the microinjection of apomorphine during the short time after surgery. It should be noted that it was a technical limitation to this work. Considering the heterogeneity of the medial prefrontal cortex, it is remained to determine the role of other subregions of the medial prefrontal cortex including the prelimbic and anterior cingulated on extracellular dopamine level in subcortical systems. Further research is necessary to evaluate other sub-territories and other doses of apomorphine. 

## Conclusion

This study showed that intra infralimbic apomorphine -induced climbing at dose of 5 μg/0.5 μl is not modulated via the increase of dopamine level in the nucleus accumbens area. This behavior may be a direct effect related to the modulation of dopamine receptors of the infralimbic subarea and not via the nucleus accumbens. 
